# Effect of opioid-free anesthesia on postoperative recovery in elderly patients undergoing laparoscopic abdominal surgery: a randomized controlled trial

**DOI:** 10.3389/fmed.2026.1862614

**Published:** 2026-07-22

**Authors:** Qiucheng Zhao, Yang Wang, Fujian Huang, Zhaoyang Xiao, Xiaoyu Xu

**Affiliations:** 1 Department of Anesthesiology, The Second Hospital of Dalian Medical University, Dalian, Liaoning, China; 2 Department of Anesthesiology, Affiliated Zhongshan Hospital of Dalian University, Dalian, Liaoning, China

**Keywords:** anesthesia, cholecystectomy, elderly, laparoscopy, opioid-free, postoperative recovery

## Abstract

**Background:**

Opioid-free anesthesia (OFA) has been proposed to reduce opioid-related adverse effects, although its impact on overall postoperative recovery remains uncertain. We evaluated whether OFA improves postoperative quality of recovery compared with opioid-based anesthesia (OA) in elderly patients undergoing laparoscopic abdominal surgery.

**Methods:**

In this single-center randomized controlled trial, 200 patients aged ≥60 years undergoing elective laparoscopic abdominal surgery were randomized to receive either OFA (dexmedetomidine, esketamine, and lidocaine) or conventional OA. The primary outcome was quality of recovery measured using the QoR-15 questionnaire at 24 h after surgery. Secondary outcomes included postoperative nausea and vomiting (PONV), pain scores, opioid consumption, gastrointestinal recovery, sleep quality, chronic postsurgical pain, hemodynamic events, extubation time, PACU length of stay, and hospital length of stay.

**Results:**

A total of 195 patients completed the study (OFA, n = 98; OA, n = 97). QoR-15 scores at 24 h were higher in the OFA group (110 ± 12 vs. 106 ± 15, mean difference 4 points, 95% CI 2–8; *p* = 0.002). However, the observed difference was below the prespecified threshold for clinical significance and was no longer present at 48 or 72 h. OFA reduced the incidence of PONV during the first 72 postoperative hours and modestly reduced postoperative opioid consumption. Gastrointestinal recovery occurred earlier with OFA, whereas pain scores were largely similar between groups except during the immediate PACU period. OFA was associated with longer extubation times, prolonged PACU stay, and a higher incidence of intraoperative hypertension. Hospital length of stay and postoperative delirium were similar between groups. Although chronic postsurgical pain at 3 months was less frequent in the OFA group, this secondary finding should be considered exploratory.

**Conclusion:**

In elderly patients undergoing laparoscopic abdominal surgery, OFA reduced postoperative nausea and vomiting and modestly accelerated gastrointestinal recovery. However, it did not result in a clinically meaningful improvement in overall postoperative recovery as measured by the QoR-15 and was associated with delayed emergence, prolonged PACU stay, and more frequent intraoperative hypertension. These findings suggest that the principal benefit of OFA in this setting may be reduction of opioid-related adverse effects rather than substantial enhancement of global postoperative recovery.

## 1 Introduction

Opioids remain widely used during general anesthesia but are associated with important perioperative adverse effects (1, 2). Increasing attention has therefore been directed toward the perioperative risks associated with opioid exposure. Even short-term opioid administration may cause respiratory depression and postoperative nausea and vomiting (PONV) (3). These complications may delay postoperative recovery and prolong hospitalization (4).

Opioid-free anesthesia (OFA) has been proposed as one such alternative (5). OFA typically combines non-opioid agents such as dexmedetomidine, ketamine, lidocaine, and magnesium to reduce perioperative opioid exposure (6, 7). Although OFA has gained increasing interest within enhanced recovery pathways, current evidence regarding its overall clinical benefit remains heterogeneous, particularly with respect to patient-reported recovery outcomes (8). Previous studies have suggested that OFA may reduce PONV and improve postoperative recovery (9, 10).

The majority of studies have focused on relatively young or middle-aged patients undergoing procedures such as gynecologic, orthopedic, or thoracic surgery. The elderly population, which represents a growing proportion of surgical patients worldwide, remains understudied. Elderly patients are particularly vulnerable because age-related physiological decline, frailty, and comorbidities increase sensitivity to opioid-related adverse effects (11). For example, dexmedetomidine may predispose older patients to bradycardia or hypotension, while esketamine carries a risk of psychomimetic effects. These considerations underscore the need for careful evaluation of OFA in this demographic (12).

Therefore, this randomized controlled trial aimed to evaluate whether OFA improves postoperative quality of recovery (QoR) compared with opioid-based anesthesia (OA) in elderly patients undergoing laparoscopic abdominal surgery. We hypothesized that OFA would improve QoR-15 scores at 24 h postoperatively.

## 2 Materials and methods

### 2.1 Study population

This randomized controlled trial was conducted at the Second Affiliated Hospital, Dalian Medical University, between April 2024 and October 2024. A total of 300 elderly patients scheduled for elective laparoscopic abdominal surgery were screened for eligibility in this randomized controlled trial at the Second Affiliated Hospital of Dalian Medical University (ChiCTR2400094317). Eligible patients were 60 years of age or older, classified as American Society of Anesthesiologists physical status I–III, and scheduled to undergo elective laparoscopic abdominal surgery under general anesthesia. All participants provided written informed consent before enrollment. Patients were excluded if they met any of the following conditions: severe bradycardia (resting heart rate <50 beats/min), uncontrolled hypertension (systolic blood pressure >180 mmHg or diastolic blood pressure >100 mmHg), advanced cardiac dysfunction defined as a left ventricular ejection fraction <35%, or severe hepatic impairment corresponding to Child–Pugh class C. Additional exclusion criteria included a history of chronic pain requiring long-term analgesic or sedative therapy, a known allergy or intolerance to any of the study medications, or cognitive or communication barriers that would prevent patients from reliably completing validated assessment instruments such as the visual analogue scale (VAS) and the QoR-15. Finally, patients who had participated in another interventional clinical trial within the preceding 30 days were excluded to minimize potential confounding effects.

### 2.2 Randomization and blinding

After eligibility was verified during the preoperative anesthesia consultation and informed consent had been obtained, participants were randomized in a 1:1 ratio to receive either OFA or conventional OA. To maintain allocation concealment, randomization codes were prepared in advance by an anesthesiologist who had no further involvement in perioperative management or data collection. Each assignment was sealed in a sequentially numbered, opaque envelope. On the day of surgery, immediately before induction, another anesthesiologist, who was responsible only for intraoperative care and not for postoperative evaluation, opened the envelope and implemented the assigned anesthetic protocol. Blinding was rigorously maintained. Patients, postoperative care teams, and investigators assessing outcomes were unaware of group allocation throughout the trial. Only the intraoperative anesthesiologist was aware of the treatment assignment, and this individual had no role in postoperative care or in the collection and analysis of outcomes. This approach minimized the potential for bias and preserved the internal validity of the study.

### 2.3 Anesthetic management

All participants observed a fasting interval of 6–8 h and did not receive premedication. On arrival in the operating theatre, standard monitoring was established, including continuous electrocardiography, pulse oximetry, invasive or noninvasive arterial blood pressure measurement, and bispectral index (BIS) monitoring to guide anesthetic depth (13). To standardize baseline prophylaxis, all patients received intravenous flurbiprofen axetil 50 mg (Beijing Tide Pharmaceutical Co., Ltd.; Beijing, China) and dexamethasone sodium phosphate 10 mg (Yangtze River Pharmaceutical Group; Taizhou, China) after monitors were applied and before induction.

In the OFA group, patients received a loading infusion of dexmedetomidine 0.5 μg·kg^−1^ over 10 min (Hengrui Medicine Co., Ltd.; Jiangsu, China) before induction. Anesthesia was then induced with propofol 1.5–2.5 mg·kg^−1^ (Fresenius Kabi; Bad Homburg, Germany), lidocaine 1.5 mg·kg^−1^ (Shandong Hualu Pharmaceutical Co., Ltd.; Shandong, China), remazolam 0.1 mg·kg^−1^ (Hengrui Medicine Co., Ltd.; Jiangsu, China), rocuronium 0.6 mg·kg^−1^ (Merck Sharp & Dohme; Rahway, NJ, United States), and esketamine 0.5 mg·kg^−1^ (Hengrui Medicine Co., Ltd.; Jiangsu, China). Maintenance consisted of continuous infusions of dexmedetomidine 0.3 μg·kg^−1^·h^−1^, lidocaine 1.5 mg·kg^−1^·h^−1^, and esketamine 0.3 mg·kg^−1^·h^−1^, in combination with sevoflurane (Hengrui Medicine Co., Ltd.; Jiangsu, China) adjusted to maintain BIS values between 40 and 60.

Intra-operative analgesic adequacy was assessed using routine clinical parameters, including heart rate, mean arterial pressure (MAP), and BIS, as dedicated nociception monitoring devices (e.g., surgical pleth index or nociception level index) were not available during the study period. Transient increases in heart rate (>10 beats/min) or MAP (>15% above the individual pre-incision baseline) were evaluated by the attending anesthesiologist. If judged to reflect insufficient analgesia or sympathetic activation, anesthetic depth or vasoactive medications were adjusted accordingly. Therefore, although background infusion rates were predefined, intraoperative management was not completely fixed and allowed clinical titration based on hemodynamic responses.

Intraoperative hypertension was defined as an increase in MAP ≥20% above the individual pre-induction baseline sustained for at least 3 consecutive minutes or requiring pharmacologic treatment. Hemodynamic variables were recorded at 5-min intervals. When this threshold was reached, vasoactive medication was administered according to institutional protocol.

Patients in the OA group received induction with propofol 1.5–2.5 mg·kg^−1^ (Fresenius Kabi; Germany), remazolam 0.1 mg·kg^−1^ (Humanwell; China), rocuronium 0.6 mg·kg^−1^ (MSD; United States), and sufentanil 0.3 μg·kg^−1^ (Yichang Humanwell Pharmaceutical Co., Ltd.; Yichang, China). Maintenance was achieved with remifentanil 0.15 μg·kg^−1^·min^−1^ (Yichang Humanwell Pharmaceutical Co., Ltd.; Yichang, China) and sevoflurane titrated to BIS 40–60. Following tracheal intubation, all patients received ultrasound-guided bilateral transversus abdominis plane block and posterior rectus sheath blocks using 0.25% ropivacaine (Qilu Pharmaceutical Co., Ltd.; Jinan, China). KY2024 For each transversus abdominis plane block, 20 mL of 0.25% ropivacaine was injected per side between the internal oblique and transversus abdominis muscles. For each posterior rectus sheath block, 10 mL per side was administered under ultrasound guidance.

Lung-protective ventilation was employed with tidal volumes of 6–8 mL·kg^−1^ predicted body weight and end-tidal carbon dioxide maintained between 35 and 40 mmHg. Normothermia (36–37 °C) was preserved with a forced-air warming blanket, and crystalloids or colloids were administered at the discretion of the attending anesthesiologist. Vasoactive medications were used as required to maintain hemodynamic stability. At the conclusion of surgery, all patients received intravenous ramosetron 0.3 mg (TongDe Pharmaceutical Co., Ltd.; Chengdu, China) for prophylaxis against PONV. Postoperative analgesia was provided with a patient-controlled analgesia pump containing sufentanil 150 μg (Humanwell; China) and ramosetron 0.6 mg (Astellas; Japan), diluted in normal saline to 150 mL (final sufentanil concentration 1 μg·mL^−1^). A low-dose background infusion was included in the patient-controlled analgesia protocol according to institutional practice for elderly patients. The same regimen was applied to both groups to ensure consistency of postoperative analgesia. The patient-controlled analgesia was programmed with a background infusion of 3 mL·h^−1^, a bolus dose of 3 mL, and a lockout interval of 30 min. In the post-anesthesia care unit (PACU) (14), breakthrough pain (VAS ≥ 4) was treated with incremental sufentanil 5 μg IV boluses (minimum 10-min intervals), while severe nausea or vomiting was managed with metoclopramide 10 mg IV (KaiFeng Pharmaceutical Co., Ltd.; Kaifeng, China).

### 2.4 Outcomes

Outcomes were selected to capture both early postoperative recovery and longer-term patient-centered endpoints, providing a comprehensive evaluation of the anesthetic strategies. The primary outcome was the QoR-15 score at 24 h after surgery (postoperative day 1, POD1). QoR-15 scores measured on postoperative days 2 and 3 were predefined secondary outcomes. The QoR-15 evaluates multiple domains, including physical comfort, emotional well-being, physical independence, psychological support, and pain, yielding a total score between 0 and 150, with higher scores reflecting superior recovery (15). Secondary outcomes encompassed several short-term clinical parameters: length of stay in the PACU (14) and overall hospital stay; incidence of PONV within the first three postoperative days; postoperative pain intensity assessed by the VAS; and cumulative sufentanil consumption. Additional measures included perioperative hemodynamic stability, gastrointestinal recovery defined as the time to first flatus, occurrence of POD assessed with the Confusion Assessment Method for the Intensive Care Unit, and postoperative sleep quality evaluated with the Richards-Campbell Sleep Questionnaire (RCSQ). Long-term outcomes were evaluated 3 months after surgery by structured telephone follow-up conducted by trained investigators. Chronic postsurgical pain (CPSP) was assessed at 3 months postoperatively using the Brief Pain Inventory, and sleep quality was evaluated using the Pittsburgh Sleep Quality Index (PSQI). CPSP was defined as pain persisting for at least 3 months after surgery that was different from preoperative pain and could not be explained by other causes or pre-existing pain conditions (16). Follow-up assessments were conducted using structured telephone interviews performed by trained investigators blinded to group allocation. During the interview, patients were asked about the presence, location, and intensity of persistent pain, as well as current analgesic use. No in-person physical examination or quantitative sensory testing was performed.

### 2.5 Hemodynamic monitoring

Hemodynamic data were systematically collected at seven predefined perioperative time points to ensure consistency across patients: baseline in the supine resting state upon entry to the operating room (T0), immediately after anesthetic induction (T1), following tracheal intubation (T2), at the time of skin incision (T3), at the initiation of pneumoperitoneum (T4), after surgery (T5), and at the termination of anesthesia (T6). Continuous monitoring of MAP and heart rate was performed using invasive radial arterial lines whenever possible; in patients where invasive access was not feasible, validated noninvasive arterial pressure monitoring was employed. All monitors were calibrated and zeroed before induction to reduce measurement bias.

Blood pressure variability (BPV) was analyzed to provide a more nuanced description of intraoperative hemodynamic stability beyond isolated MAP or heart rate values. Variability was first expressed as the standard deviation (SD) of MAP and heart rate during the study period. To account for inter-individual differences in baseline pressure, relative variability was calculated using the coefficient of variation (CV), defined as the SD divided by the mean value. Sequential fluctuations were further assessed by average real variability (ARV), which represents the mean absolute difference between consecutive MAP readings across time points. This measure captures short-term oscillations that may be clinically significant but masked by average values.

By combining absolute, relative, and sequential indices, BPV analysis provided a comprehensive characterization of perioperative cardiovascular responses under the two anesthetic regimens. These parameters were selected because greater intraoperative BPV has been linked to adverse outcomes such as myocardial injury, renal dysfunction, and delayed recovery, making them clinically relevant surrogate endpoints in elderly surgical patients.

### 2.6 Sample size calculation

The sample size was calculated *a priori* based on the primary endpoint, the QoR-15 score at 24 h postoperatively. Previous studies of recovery after laparoscopic surgery suggested that an 8-point difference in QoR-15 score, with an assumed SD of 15 points, would represent a clinically meaningful improvement. Assuming a two-sided *α* of 0.05 and a power of 80%, 90 patients were required per group using a two-sample *t*-test. To account for an anticipated dropout rate of 10%, the final target enrollment was set at 200 patients (100 per group).

### 2.7 Statistical analysis

All statistical analyses were performed using IBM SPSS Statistics, version 22.0 (IBM Corp., Armonk, NY, United States), according to the intention-to-treat principle, including all randomized patients. The distribution of continuous variables was tested using the Shapiro–Wilk test. Normally distributed continuous variables are presented as mean ± SD, non-normally distributed variables are presented as median [interquartile range, IQR]. Categorical variables are expressed as counts and percentages and were compared using the chi-square test or Fisher’s exact test, as appropriate. Effect estimates are presented as mean differences with 95% confidence intervals (CI) for continuous variables and relative risks (RR) with 95% CI for binary outcomes. CI for RR were calculated using the log transformation method. A two-sided *p* value < 0.05 was considered statistically significant. No formal adjustment for multiplicity was performed for secondary outcomes; these analyses should therefore be interpreted as exploratory.

## 3 Results

### 3.1 Enrollment and baseline characteristics

**Figure 1:**
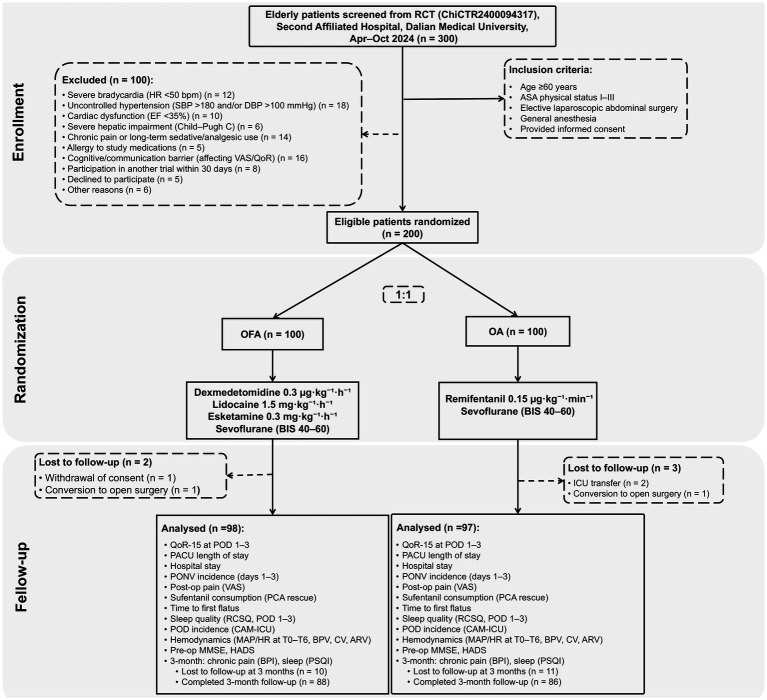
CONSORT flow diagram of the study. Elderly patients undergoing elective laparoscopic abdominal surgery were assessed for eligibility at the Second Affiliated Hospital of Dalian Medical University between April 2024 and October 2024. Of 300 patients screened, 200 were randomized in a 1:1 ratio to receive opioid-free anesthesia (OFA) or opioid-based anesthesia (OA). Participant progression, exclusions, and follow-up are detailed in the diagram. A total of 98 patients in the OFA group and 97 patients in the OA group were included in the final analysis. OFA, opioid-free anesthesia; OA, opioid anesthesia; HR, heart rate; SBP, systolic blood pressure; DBP, diastolic blood pressure; EF, ejection fraction; ASA, American Society of Anesthesiologists physical status; BIS, bispectral index; PACU, post-anesthesia care unit; PONV, postoperative nausea and vomiting; VAS, visual analogue scale; QoR-15, 15-item Quality of Recovery questionnaire; RCSQ, Richards–Campbell Sleep Questionnaire; CAM-ICU, Confusion Assessment Method for the Intensive Care Unit; MMSE, Mini-Mental State Examination; HADS, Hospital Anxiety and Depression Scale; BPI, Brief Pain Inventory; PSQI, Pittsburgh Sleep Quality Index.

A total of 300 patients were screened, of whom 200 were randomized. The detailed study flow is presented in Figure 1. Baseline demographic, clinical, and surgical characteristics were comparable between the OFA and OA groups (Table 1). Age distribution, sex proportion, body mass parameters, and major comorbidities were similar between groups. The distribution of surgical procedures and ASA physical status showed no meaningful differences. Preoperative assessments, including sleep quality, cognitive screening, and psychological status, were also balanced, indicating adequate comparability at enrollment.

**Table 1:** Baseline demographic and clinical characteristics of patients in the OFA and OA groups.

Characteristic	OFA group (*n* = 98)	OA group (*n* = 97)	*p*-value
Demographics			
Age, years (mean ± SD)	68.97 ± 5.75	70.72 ± 7.35	0.065
Female sex, *n* (%)	33 (33.7)	36 (37.1)	0.209
Weight, kg (mean ± SD)	66.0 ± 9.78	68.28 ± 11.01	0.127
Height, cm (mean ± SD)	166.39 ± 7.15	166.65 ± 8.18	0.812
BMI, kg/m^2^ (mean ± SD)	23.84 ± 3.58	24.59 ± 3.89	0.161
Medical history, *n* (%)			
Hypertension	35 (35.7)	41 (42.3)	0.429
Diabetes mellitus	23 (23.5)	23 (23.7)	0.968
Pulmonary disease	3 (3.1)	3 (3.1)	0.990
Cerebrovascular disease	6 (6.1)	9 (9.4)	0.396
Smoker	38 (38.8)	34 (35.1)	0.590
History of nausea/vomiting	7 (7.1)	10 (10.3)	0.433
History of car sickness	19 (19.6)	16 (16.7)	0.598
Type of surgery, *n* (%)			
Gastric carcinoma	39 (39.8)	32 (33.0)	0.217
Colorectal cancer	51 (52.0)	60 (61.9)	
Prostatic cancer	4 (4.1)	5 (5.2)	
Hepatic and biliary surgery	3 (3.1)	0 (0.0)	
Gynecological	1 (1.0)	0 (0.0)	
ASA physical status, *n* (%)			
II	72 (73.5)	66 (68.0)	0.405
III	26 (26.5)	31 (32.0)	
Preoperative scores, median (IQR)			
PSQI score	7 (3)	7 (5)	0.600
Cognitive screening score	2 (1)	2 (1)	0.685
HADS score	3 (2)	3 (2)	0.556

Values are presented as mean ± standard deviation (SD), median (interquartile range [IQR]), or number (%). ASA, American Society of Anesthesiologists; HADS, Hospital Anxiety and Depression Scale; IQR, interquartile range; OA, opioid-based anesthesia; OFA, opioid-free anesthesia; PSQI, Pittsburgh Sleep Quality Index; SD, standard deviation.

### 3.2 Quality of recovery and analgesia

The primary endpoint, QoR-15 at 24 h after surgery (POD1), was significantly higher in the OFA group compared with the OA group (110 ± 12 vs. 106 ± 15; mean difference 4 points, 95% CI: 2 to 8; *p* = 0.002; Table 2). At 48 and 72 h, QoR-15 scores were comparable between groups (48 h: 113 ± 13 vs. 114 ± 16; *p* = 0.334; 72 h: 121 ± 10 vs. 120 ± 13; *p* = 0.257; Table 2), indicating that the early recovery advantage observed at 24 h did not persist beyond the first postoperative day. Although statistically significant, the between-group difference at 24 h was smaller than the 8-point difference prespecified in the sample size calculation. Therefore, while OFA was associated with modest early improvement in recovery, the clinical relevance of this effect should be interpreted with caution.

**Table 2:** Quality of recovery (QoR-15) scores at 24, 48, and 72 h after surgery.

Time point	OFA (*n* = 98)	OA (*n* = 97)	Mean difference (95% CI)	*P* value
24 h†	110 ± 12	106 ± 15	4 (2 to 8)	0.002
48 h	113 ± 13	114 ± 16	−1 (−2 to 5)	0.334
72 h	121 ± 10	120 ± 13	1 (−1 to 4)	0.257

Values are presented as mean ± standard deviation (SD). † QoR-15 at 24. OFA, opioid-free anesthesia; OA, opioid-based anesthesia; QoR-15, 15-item Quality of Recovery questionnaire; CI, confidence interval; SD, standard deviation h was the prespecified primary endpoint.

### 3.3 Short-term recovery finding

Short-term recovery outcomes differed between the two anesthetic strategies (Table 3). Analgesic requirements were lower in patients receiving OFA. Mean sufentanil consumption was significantly reduced at both 24 h (72 ± 31.0 μg vs. 74.9 ± 32.4 μg; mean difference −2.9 μg [95% CI: −5.47 to −0.33]; *p* = 0.032) and 48 h (144 ± 35.9 μg vs. 162 ± 30.7 μg; mean difference −18.0 μg [95% CI: −27.43 to −8.57]; *p* = 0.010). Pain intensity measured by VAS remained broadly comparable over the first three postoperative days. Notably, however, OFA patients reported less pain immediately after surgery in the PACU, both at rest (median 0 [IQR 2] vs. 2 [1]; *p* < 0.001) and on coughing (1 [2] vs. 2 [1]; p < 0.001).

**Table 3:** Short-term postoperative outcomes indices in OFA and OA groups.

Outcome	OFA (n = 98)	OA (*n* = 97)	Effect estimate (95% CI)	*P* value
Analgesia (μg)				
Sufentanil consumption, 24 h	72 ± 31.0	74.9 ± 32.4	−2.9 (−11.5 to 5.7)	0.032*
Sufentanil consumption, 48 h	144 ± 35.9	162 ± 30.7	−18.0 (−27.4 to −8.6)	0.010*
Pain (VAS) (median [IQR])				
PACU, rest	0 [2]	2 [1]	–	<0.001*
PACU, cough	1 [2]	2 [1]	–	<0.001*
POD1–3 (rest/cough)*^#^*	Similar	Similar	–	NS
PONV incidence (*n*, %)			RR (95% CI)	
PACU	0 (0%)	1 (1.0%)	–	0.497
24 h	23 (23.5%)	53 (54.6%)	0.43 (0.29–0.64)	<0.001*
48 h	14 (14.3%)	34 (35.1%)	0.41 (0.23–0.71)	0.001*
72 h	6 (6.1%)	19 (19.6%)	0.31 (0.13–0.75)	0.005*
Recovery times (median [IQR])				
Extubation (min)	26 [20]	10 [10]	+16 min	<0.001*
PACU stay (min)	60 [30]	40 [18]	+20 min	<0.001*
Time to flatus (h)	47 [34]	56 [31]	–9 h	0.045*
Hospital stay (days)	8 [2]	8 [2]	0	0.400
Postoperative delirium (*n*, %)	1 (1.0%)	1 (1.0%)	RR 1.00	1.000
Sleep quality (RCSQ) (mean ± SD)				
RCSQ 24 h	51.8 ± 36.8	33.9 ± 36.1	+17.9 (7.60 to 28.20)	0.012*
RCSQ 48 h	61.3 ± 22.9	58.2 ± 26.8	+3.1 (−3.95 to 10.15)	0.112
RCSQ 72 h	63 ± 21.7	57.7 ± 16.6	+5.3 (−0.16 to 10.76)	0.062
Intraoperative hypertension (n, %)	21 (21.6%)	7 (7.2%)	3.00 (1.13–6.75)	0.004
Perioperative characteristics (median [IQR])				
Surgery duration (min)	170 [71]	185 [60]	−15 min	0.319
Anesthesia duration (min)	206 [117]	205 [65]	+1 min	0.989
Fluid infused (mL)	1800 [800]	1700 [600]	+100 mL	0.934
Urine output (mL)	370 [258]	450 [440]	−80 mL	0.070
Intraoperative adverse events (*n*, %)^##^			RR (95% CI)	
Hypertension	21 (21.6%)	7 (7.2%)	2.97 (1.32–6.66)	0.004*
Hypotension	37 (37.8%)	34 (35.1%)	1.08 (0.75–1.55)	0.695
Bradycardia	9 (9.2%)	4 (4.1%)	2.23 (0.71–6.99)	0.157
Tachycardia	4 (4.1%)	4 (4.1%)	0.99 (0.25–3.85)	1.000

Values are expressed as mean ± standard deviation (SD), median [interquartile range, IQR], or number (percentage). OFA, opioid-free anesthesia; OA, opioid-based anesthesia; VAS, visual analogue scale; PACU, post-anesthesia care unit; PONV, postoperative nausea and vomiting; POD, postoperative day; RCSQ, Richards–Campbell Sleep Questionnaire; NS, not significant; RR, relative risk; CI, confidence interval; RR, relative risk; CI, confidence interval. No adjustment for multiplicity was performed for secondary outcomes. * *P* < 0.05 indicates statistical significance. # POD1–3 (rest/cough) indicates VAS pain scores assessed at rest and during coughing on postoperative days 1–3. ## Intraoperative hemodynamics were guided by heart rate, mean arterial pressure, and bispectral index according to institutional protocol.

OFA was associated with a lower incidence of PONV. Compared with OA, PONV incidence was lower at 24 h (23.5% vs. 54.6%; RR 0.43 [95% CI: 0.29–0.64]; *p* < 0.001), 48 h (14.3% vs. 35.1%; RR 0.41 [95% CI: 0.23–0.71]; *p* = 0.001), and 72 h (6.1% vs. 19.6%; RR 0.31 [95% CI: 0.13–0.75]; *p* = 0.005). Gastrointestinal recovery occurred earlier in the OFA group, with a shorter median time to first passage of flatus (47 h vs. 56 h; difference −9 h; *p* = 0.045). In contrast, emergence from anesthesia was delayed with OFA. Extubation time was longer compared with OA (26 min [IQR 20] vs. 10 min [IQR 10]; difference +16 min; *p* < 0.001), and discharge from the PACU was also prolonged (60 min [IQR 30] vs. 40 min [IQR 18]; difference +20; p < 0.001). Length of hospital stay was not different between groups (median 8 days in both; *p* = 0.400). POD occurred rarely and at the same frequency in both groups (1.0% vs. 1.0%; 1% each). Patients in the OFA group demonstrated higher RCSQ scores on the first postoperative night (51.8 ± 36.8 vs. 33.9 ± 36.1; mean difference +17.9 [95% CI: 7.60–28.20]; *p* = 0.012). By 48 and 72 h, sleep quality scores were similar between groups, and no statistically significant differences were observed (*p* > 0.05). Surgical duration, anesthetic duration, fluid infusion volumes, and urine output were comparable between groups. Intraoperative hypertension occurred more frequently in the OFA group despite protocol-based hemodynamic management guided by standard clinical parameters. Intraoperative hypertension, defined according to the predefined MAP criteria, occurred more frequently in the OFA group (21.6% vs. 7.2%; RR 2.97 [95% CI: 1.32–6.66]; *p* = 0.004). The incidences of hypotension, bradycardia, and tachycardia were similar between groups and did not differ significantly.

### 3.4 Long-term outcomes and hemodynamics

**Figure 2:**
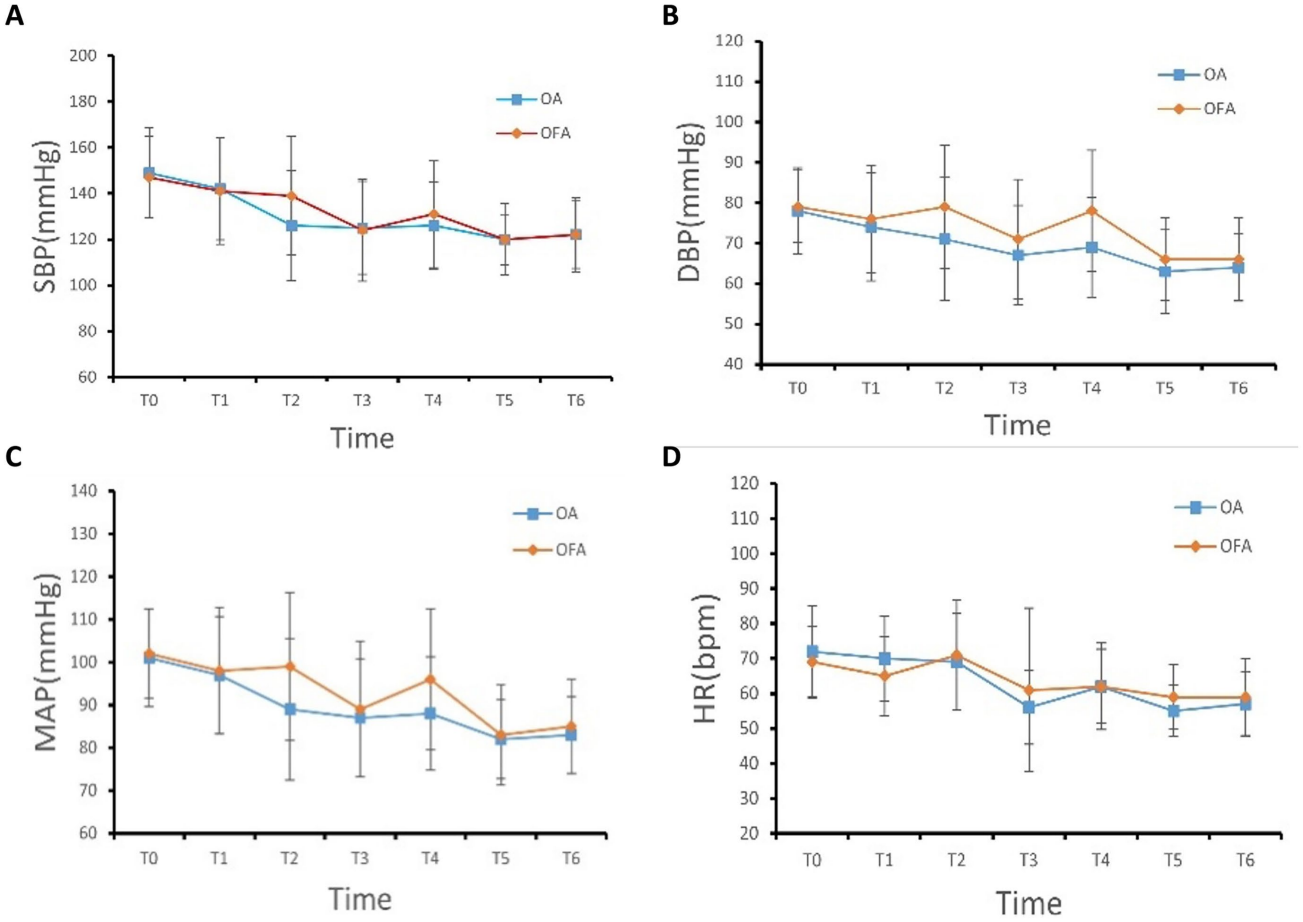
Intraoperative hemodynamic data. **(A)** Systolic blood pressure, **(B)** diastolic blood pressure, **(C)** mean arterial pressure, and **(D)** heart rate are shown for OFA and OA groups at key perioperative timepoints: T0, baseline; T1, post-induction; T2, post-intubation; T3, skin incision; T4, pneumoperitoneum initiation; T5, surgical conclusion; T6, anesthesia termination. Both groups demonstrated the expected sequence of perioperative fluctuations, early decreases after induction and intubation, transient elevations with incision and pneumoperitoneum, and recovery by the end of surgery. No clinically meaningful differences were observed between groups across any parameter, confirming comparable hemodynamic stability under both anesthetic strategies.

At 3 months postoperatively, follow-up data were available for 88 patients in the OFA group and 86 patients in the OA group (Table 4). CPSP occurred in 11 of 88 patients (12.5%) in the OFA group and 29 of 86 patients (33.7%) in the OA group (RR 0.37, 95% CI: 0.20–0.69; *p* = 0.001). Sleep quality at 3 months, assessed using the PSQI, was lower in the OFA group compared with the OA group (6 [IQR 3] vs. 8 [IQR 5]; *p* < 0.001) (Table 4). Hemodynamic variability indices, including BPV, CV, and ARV for systolic, diastolic, and MAP, did not differ significantly between groups (Table 4). Intraoperative blood pressure and heart rate trajectories were comparable between groups (Figure 2).

**Table 4:** Long-term outcomes and hemodynamic variability indices in OFA and OA groups.

Outcome	OFA	OA	Effect estimate (95% CI)	*P* value
Three-month outcomes				
CPSP, *n*/*N* (%)	11/88 (12.5%)	29/86 (33.7%)	RR 0.37 (0.20–0.69)	0.001*
PSQI score (median [IQR])	6 [3]	8 [5]	–	<0.001*
Hemodynamic variability indices (median [IQR])				
BPV-SBP	16.5 [8.0]	17.0 [9.5]	–	0.915
CV-SBP	0.13 [0.05]	0.13 [0.06]	–	0.734
ARV-SBP	17.4 [8.3]	16.2 [6.7]	–	0.095
BPV-DBP	10.0 [5.0]	10.0 [5.0]	–	0.439
CV-DBP	0.14 [0.06]	0.13 [0.06]	–	0.827
ARV-DBP	11.1 [5.9]	9.3 [6.0]	–	0.053
BPV-MAP	12.0 [6.0]	11.0 [6.5]	–	0.992
CV-MAP	0.13 [0.07]	0.13 [0.06]	–	0.558
ARV-MAP	12.0 [7.4]	10.8 [6.3]	–	0.261

Values are expressed as median [interquartile range, IQR] or *n*/*N* (%). OFA, opioid-free anesthesia; OA, opioid-based anesthesia; CPSP, chronic postsurgical pain; PSQI, Pittsburgh Sleep Quality Index; BPV, blood pressure variability; CV, coefficient of variation; ARV, average real variability; SBP, systolic blood pressure; DBP, diastolic blood pressure; MAP, mean arterial pressure; RR, relative risk; CI, confidence interval. * *P* < 0.05 indicates statistical significance.

### 3.5 Gastrointestinal and overall recovery trajectories

**Figure 3:**
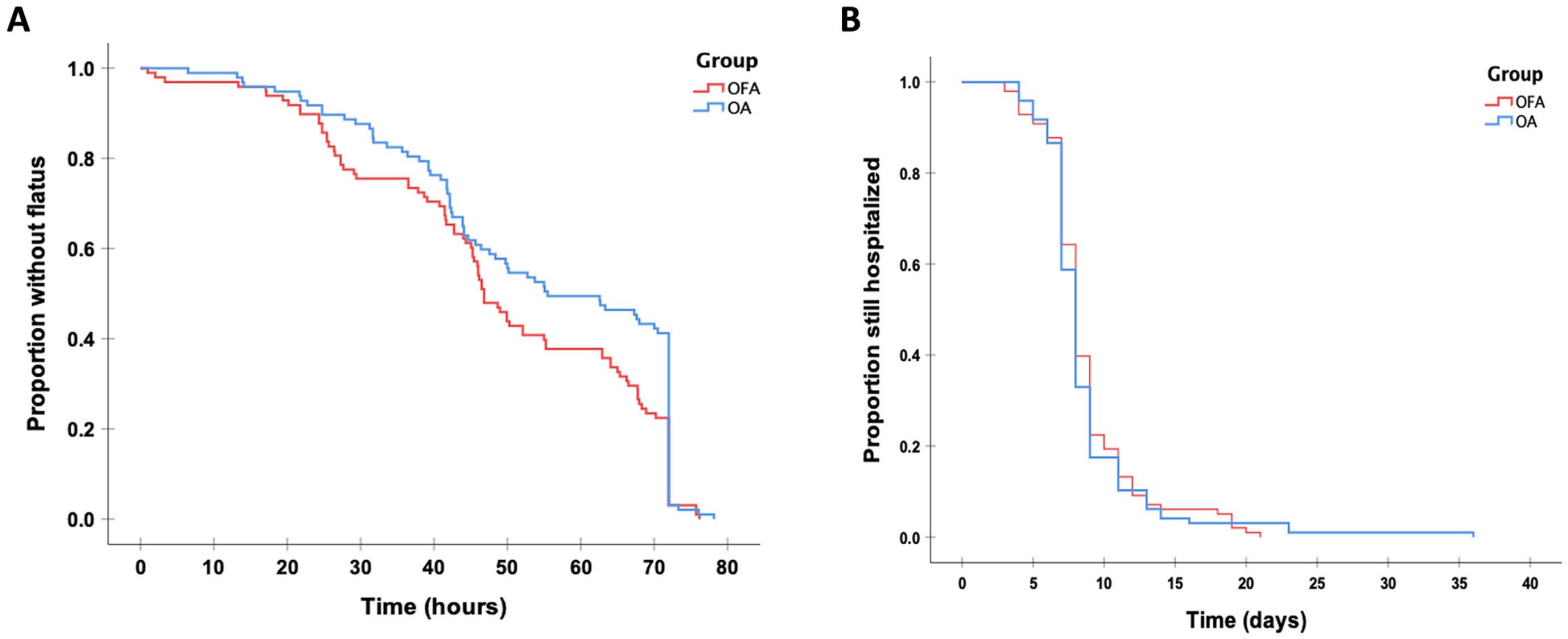
Kaplan–Meier curves for postoperative recovery. **(A)** Time to first flatus (hours). **(B)** Time to hospital discharge (days). Curves are stratified by anesthesia group. The red line represents the OFA group, and the blue line represents the OA group. The median time to first flatus was 46.8 h (95% CI: 43.6–50.1) in the OFA group and 55.5 h (95% CI: 40.0–71.0) in the OA group (*p* = 0.019, log-rank test). No significant difference was observed in hospital length of stay between the two groups (*p* = 0.715, log-rank test).

Kaplan–Meier analysis demonstrated a shorter time to first flatus in the OFA group compared with the OA group (46.8 h [95% CI: 43.6–50.1] vs. 55.5 h [95% CI: 40.0–71.0]; log-rank *p* = 0.019) (Figure 3). The curves separated early after surgery, reflecting faster gastrointestinal recovery in the OFA group. No significant difference was observed in the length of hospital stay between groups (log-rank *p* = 0.715).

## 4 Discussion

OFA clearly reduces postoperative nausea and vomiting in elderly patients, but does not provide a clinically meaningful improvement in overall recovery as measured by the QoR-15. These modest benefits came at the cost of delayed emergence and a higher incidence of intraoperative hypertension. Overall, the observed advantages appear limited and are driven primarily by the antiemetic effects of opioid avoidance. The present trial demonstrated that OFA was associated with several statistically significant perioperative benefits in elderly patients undergoing laparoscopic abdominal surgery, particularly reduced PONV and earlier gastrointestinal recovery. However, the observed improvement in QoR-15 at 24 h was modest, did not reach the prespecified clinically meaningful threshold used for sample size estimation, and was not sustained beyond the first postoperative day. Therefore, the overall clinical significance of the recovery benefit should be interpreted cautiously.

The improvement in QoR-15 scores at 24 h in the OFA group is consistent with prior studies demonstrating that this multidimensional patient-reported outcome measure is sensitive to early recovery differences (17–20). Léger et al. (19) showed higher QoR-15 scores in patients receiving OFA during major surgery, Zhang et al. (20) reported improved recovery in breast surgery, and Hao et al. (17) confirmed its benefit in laparoscopic cholecystectomy. Our results align with these observations but extend them to an elderly cohort, which is particularly relevant given the higher vulnerability of older patients to anesthetic side effects. Importantly, the intergroup difference in QoR-15 scores in our study did not persist at 48 or 72 h (5). This convergence may reflect the influence of standardized postoperative care, multimodal analgesia, and enhanced recovery measures, which likely minimized the impact of anesthetic strategy beyond the first day. In addition, elderly patients often recover more slowly due to age-related physiological changes and comorbidities, which may have further diluted the early benefits of OFA (21). Importantly, although the primary endpoint reached statistical significance, the observed between-group difference in QoR-15 scores did not achieve the prespecified MCID of 8 points. Therefore, while OFA may confer some recovery-related benefit, the magnitude of clinical improvement remains uncertain. These findings suggest that the superiority of OFA over conventional opioid anesthesia in elderly patients should be interpreted cautiously.

Opioid consumption was significantly reduced in the OFA group at both 24 and 48 h, even though pain intensity scores were broadly comparable between groups except during the immediate PACU period (22). This observation mirrors the findings of An et al. (23), who reported lower postoperative opioid requirements but similar pain scores with OFA in laparoscopic colectomy. The apparent dissociation between opioid use and reported pain can be explained by the analgesic and anti-hyperalgesic properties of dexmedetomidine and ketamine, both of which extend their effects into the early postoperative period (24). Our results differ from those of Perez et al. (25), who found no opioid-sparing effect with an OFA regimen in bariatric surgery, suggesting that surgical type, OFA drug combination, and patient population strongly influence outcomes.

A particularly robust benefit of OFA in this trial was the modest reduction in PONV, which persisted across all three postoperative days. This is consistent with the literature showing that opioids are among the strongest risk factors for PONV (26, 27). Ziemann-Gimmel et al. (28) observed a striking reduction in PONV when opioids and volatile anesthetics were avoided in bariatric surgery. Similar findings have been reported in shoulder (29) and thoracic (30) surgery, and meta-analyses have confirmed that OFA lowers the incidence of PONV (31, 32). Because PONV is often perceived as more distressing than pain by patients, this reduction represents a tangible improvement in patient-centered recovery.

Gastrointestinal recovery is another important dimension of ERAS. Our Kaplan–Meier analysis confirmed that OFA significantly shortened the time to first flatus, with a median of 46.8 h compared with 55.5 h in the OA group, consistent with the findings of Chen et al. (33) in gynecological laparoscopy. The avoidance of opioids, which inhibit gut motility through *μ*-receptor mechanisms, together with the potential pro-motility effects of lidocaine, likely explain this difference. However, despite earlier bowel function, the length of hospital stay did not differ between groups. This discrepancy probably reflects the multifactorial determinants of discharge in elderly surgical patients, including comorbidity management, surgical factors, and institutional discharge policies, which may overshadow the contribution of anesthesia regimen (34, 35).

Our trial also demonstrated a significant reduction in CPSP at 3 months in the OFA group. This finding aligns with mechanistic evidence that perioperative opioid exposure may contribute to central sensitization and hyperalgesia (36, 37). The protective effect observed in our study adds to the growing body of evidence that anesthetic technique may have long-term consequences for pain outcomes. However, given the single-center design, limited sample size, and telephone-based follow-up methodology, these findings should be interpreted cautiously and considered exploratory. Larger multicenter studies with standardized long-term pain assessment are needed to confirm this association.

Sleep quality, an often under-reported but clinically important outcome, was improved on the first postoperative night in the OFA group. This is biologically plausible given that dexmedetomidine mimics natural sleep architecture and increases REM sleep (38), while opioids are known to disrupt both slow-wave and REM sleep (39). The benefit was transient, as differences were no longer significant after the first night, suggesting that environmental factors, pain, and hospital routines quickly become the dominant determinants of sleep quality after surgery (40).

With respect to hemodynamic stability, our analysis showed no significant differences in blood pressure indices (BPV, CV, ARV) or heart rate between groups, indicating broadly comparable hemodynamic variability overall, despite a higher incidence of intraoperative hypertension in the OFA group. OFA was associated with prolonged extubating time and extended PACU stay, which may represent clinically relevant disadvantages in elderly surgical patients. Although these delays were not associated with longer hospital stay or increased POD, they may have implications for PACU workflow, staffing, and perioperative resource utilization. These effects are likely related to the sedative properties of dexmedetomidine and esketamine and should be considered when evaluating the overall risk–benefit profile of OFA protocols (41). Nevertheless, intraoperative hypertension was more frequent in the OFA group. This apparent paradox may be explained by the sympathomimetic properties of ketamine, which can potentiate hypertensive episodes during intense surgical stimuli such as pneumoperitoneum, while dexmedetomidine, despite its sympatholytic effects, may not have fully counterbalanced these surges. Previous large trials have raised concerns about OFA-related bradycardia and hypoxemia when high doses of dexmedetomidine were used (6). In contrast, our regimen did not increase bradycardia but did increase hypertension, underscoring the need to optimize dosing strategies to balance efficacy and safety. These results reinforce the view that OFA protocols are heterogeneous in composition and effect (28, 30, 42). While the benefits of opioid avoidance on PONV and bowel function are consistent across studies, results on pain control, opioid sparing, and hemodynamic safety vary substantially (43). This heterogeneity likely reflects differences in drug choice, dosing, surgical type, and patient population. Our trial contributes novel evidence by focusing on an elderly cohort, an understudied population in prior OFA research, and by employing the QoR-15 instrument, which offers a holistic assessment of recovery beyond analgesia.

Recent literature highlights a persistent gap between controlled trials and real-world implementation of OFA, particularly regarding variability in definitions, protocols, and clinical adoption. Large European survey data demonstrate marked heterogeneity in clinical practice, with no standardized approach and substantial variation in clinician attitudes and implementation patterns, suggesting that OFA remains a heterogeneous concept rather than a uniform clinical protocol (44, 45).

From a mechanistic perspective, studies indicate that when nociception-guided monitoring is applied, outcome differences between opioid-free and opioid-sparing techniques may be attenuated, suggesting that monitoring strategy and protocol composition significantly influence observed effects (46, 47).

Consistently, systematic reviews show that while OFA reduces PONV, it does not provide clinically meaningful improvements in pain, opioid consumption, or overall recovery, with substantial heterogeneity across studies (48, 49). Collectively, these findings support the view that the main reproducible benefit of OFA is antiemesis, while broader clinical advantages remain limited (47). The present study largely confirms that the most reproducible benefit of OFA remains reduction in PONV, whereas evidence for meaningful improvements in overall recovery remains limited. Taken together, this evidence suggests that future progress will likely depend on standardized protocols and more individualized, patient-centered perioperative strategies rather than a uniform OFA approach.

The intervention evaluated in this study represents one specific multimodal OFA protocol and should not be considered representative of all OFA strategies. Because both groups also received regional anesthesia and postoperative opioid-based patient-controlled analgesia, the independent effect of intraoperative opioid avoidance cannot be completely separated from the overall multimodal analgesic approach. In addition, considerable variability exists among currently used OFA protocols. Therefore, the present findings should be interpreted within the context of the specific perioperative regimen used in this trial and should not be generalized broadly to all OFA techniques (5, 6).

Several limitations should be acknowledged. First, objective nociception monitoring devices were not used during anesthesia management. Intraoperative analgesic adequacy was therefore inferred from conventional hemodynamic and BIS parameters, which may not reliably reflect nociceptive balance in elderly patients and may have confounded interpretation of pain-related outcomes. In addition, cumulative hypertension burden, including duration above threshold or area-under-the-curve analyses, could not be reliably assessed because continuous intraoperative blood pressure recordings were not available for all patients. Second, this was a single-center study conducted in a tertiary hospital, which may limit external generalizability. Psychomimetic adverse effects potentially associated with esketamine, such as hallucinations, nightmares, or dissociative symptoms, were not systematically assessed (50). Although no severe neuropsychiatric complications were clinically documented, the absence of structured assessment limits conclusions regarding neuropsychiatric safety in elderly patients. Third, surgeon assignment was not standardized, and inter-surgeon variability may have influenced postoperative recovery outcomes. Fourth, the OFA regimen in this study consisted of esketamine, dexmedetomidine, lidocaine, and transversus abdominis plane block, representing only one possible opioid-free protocol and limiting comparability with other OFA strategies. Fifth, follow-up was limited to 3 months; longer-term follow-up would allow a more comprehensive evaluation of chronic pain and functional recovery. Sixth, CPSP was assessed by a structured telephone interview without in-person physical examination or quantitative sensory testing, which may introduce ascertainment bias. Although follow-up rates were similar between groups, incomplete follow-up may affect the interpretation of long-term outcomes. In addition, multiple secondary outcomes were analyzed without formal adjustment for multiplicity, increasing the possibility of type I error. Therefore, statistically significant secondary findings should be interpreted as exploratory and hypothesis-generating rather than definitive evidence of clinical superiority. Cardiac and renal biomarkers, such as high-sensitivity troponin, were not routinely measured in asymptomatic patients; therefore, subclinical perioperative organ injury cannot be fully excluded.

## 5 Conclusion

In elderly patients undergoing laparoscopic abdominal surgery, opioid-free anesthesia reduced postoperative nausea and vomiting and modestly accelerated gastrointestinal recovery. However, it did not result in a clinically meaningful improvement in overall postoperative recovery as measured by the QoR-15 and was associated with delayed emergence, prolonged PACU stay, and more frequent intraoperative hypertension. These findings suggest that the principal benefit of OFA in this setting may be reduction of opioid-related adverse effects rather than substantial enhancement of global postoperative recovery.

## Data Availability

The original contributions presented in the study are included in the article/supplementary material, further inquiries can be directed to the corresponding author.
